# Pseudotumoral form of schistosomiasis mimicking neuroendocrine tumor: a case report and brief review of the differential diagnosis of retroperitoneal masses

**DOI:** 10.11604/pamj.2020.37.186.26344

**Published:** 2020-10-27

**Authors:** Luis Marín-Martínez, Georgios Kyriakos, David Sánchez-Gutiérrez

**Affiliations:** 1 Sección de Endocrinología y Nutrición, Hospital General Universitario Santa Lucía, Cartagena, Spain,; 2 Servicio de Anatomía Patológica, Hospital General Universitario Santa Lucía, Cartagena, Spain

**Keywords:** Retroperitoneal mass, neuroendocrine tumor, schistosomiasis, paraganglioma

## Abstract

Differential diagnosis of retroperitoneal masses may become complex and requires careful anamnesis, physical examination and several complementary tests. We present the clinical case of a male patient aged 45 years who was diagnosed with a 4cm paraaortic lesion compatible with neuroendocrine tumor in the abdominal computed tomography (CT) exam. The workup performed with SPECT-CT, somatostatin receptors scintigraphy, MIBG scintigraphy, 24-hour urine total and fractionated catecholamines and 24-hour urine 5-OH indoleacetic did not confirm the first diagnostic impression. Finally, the lesion was biopsied and presence of micro-organisms was revealed. Further exams confirmed schistosomiasis as the cause of the paraaortic lesion. Histological diagnosis can be helpful with regard to the differential diagnosis of retroperitoneal masses.

## Introduction

Retroperitoneal masses constitute a heterogeneous group of lesions, originating in the retroperitoneal space, that pose a diagnostic challenge with view to histology and malignant potential [1]. The majority of cases are malignant tumors, of which approximately 75% are mesenchymal in origin. Primary tumors of the retroperitoneum are mainly lymphoproliferative disorders, soft tissue neoplasms and germ cell tumors [2]. Neuroendocrine tumors (NETs) arising in the abdominal cavity usually originate from the gastrointestinal tract and pancreas. NETs found in the retroperitoneum are mainly metastatic. Retroperitoneal paraganglioma usually arise from the adrenal medulla (80-90%), with the remainder (10-20%) arising from the paraaortic region [3].

Schistosomiasis is a parasitic disease very common in tropical and subtropical areas, particularly in the African continent and relatively rare in western countries [4]. Its pseudotumoral form is infrequent and can clinically simulates malignant neoplasms. The preferential site is the large intestine, at the level of the sigmoid colon and of the rectum. In the retroperitoneum only one case has been described worldwide [5]. In the present report we describe the rare case of a retroperitoneal pseudotumoral form of schistosomiasis mimicking neuroendocrine tumor and we make a brief review of the differential diagnosis of retroperitoneum lesions. An informed oral consent was obtained.

## Patient and observation

**Figure 1:**
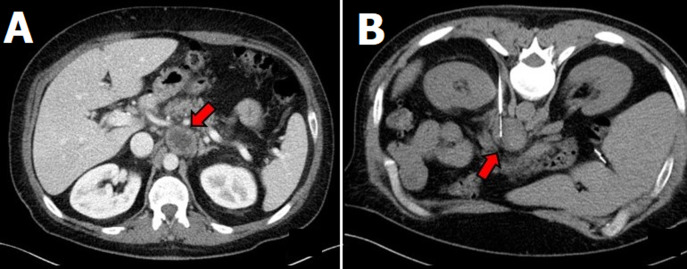
A) computed tomography (CT) abdominal scan revealing a 4cm nonspecific retroperitoneal, preaortic lesion, between celiac trunk and superior mesenteric artery; B) CT-guided biopsy of the retroperitoneal mass

**Figure 2:**
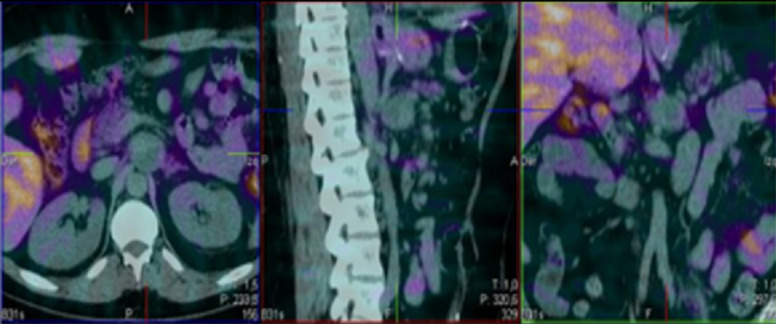
the (99m) Tc-octreotide Scintigraphy with SPECT/CT showed no evidence of pathological tumor uptake of the retroperitoneal mass

A 45-year-old Caucasian male patient was referred to our clinic of endocrinology, because of an incidental finding in a CT scan, suggestive of paraganglioma, performed in the work up for abdominal pain. The abdominal CT scan revealed a 4cm nonspecific retroperitoneal, paraaortic lesion, between celiac trunk and superior mesenteric artery, suggestive of neuroendocrine tumor as the first possibility (Figure 1A). He had a past medical history of smoking, Barret's esophagus, hiatus hernia and gastroesophageal reflux disease treated with omeprazole. He had undergone a sleeve gastrectomy as a surgical weight loss procedure in 2015 and cholecystectomy for cholelithiasis in 2017. After the first visit in our clinic, an initial laboratory investigation was performed for the suspected paraganglioma (references values in parenthesis): glucose 79 mg/dl (74-106); creatinine 0.69 mg/dl (0.70-1.30); alanine aminotransferase 22 IU/L (10-49); ferritin 73 ng/ml (22-322); enolase 19 ng/ml (<16); carcinoembryonic antigen 2.6 ng/ml (<5.0); Ca 19.9: 12 IU/ml (<37); chromogranin A: 3.3 nmol/l (0.4-10.0); hemoglobin 15.4 gr/dl (13.5-17.5); neutrophils 51.7% (40.0-70.0); lymphocytes 33.4% (22.0-44.0), eosinophils 5.3% (RV <8.0). Moreover, total and fractional catecholamines, total metanephrines and excretion of 5-OH-indolacetic in the 24-hour urine analysis were normal. Next, radioisotope imaging was ordered. The whole-body scintigraphy with metaiodobenzylguanidine didn´t reveal any clear signs suggesting the existence of catecholamine-producing tumor. The whole-body (99m) Tc-octreotide scintigraphy with SPECT/CT showed a paraaortic abdominal mass, but without evidence of pathological tumor uptake (Figure 2).

**Figure 3:**
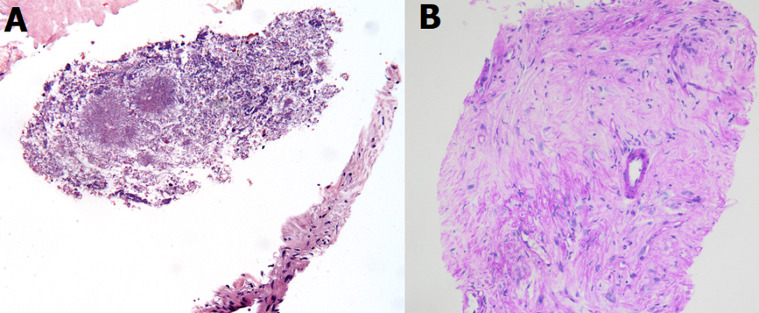
A) image of the microscopy of the biopsy suggestive of microorganisms; B) PAS-stain

After multidisciplinary discussion, it was decided to perform a CT-guided biopsy of the lesion (Figure 1B). Microscopic study of the sample demonstrated no atypia but a tissue with fibrosis, collagenization and presence of spongy-looking structures, suggestive of microorganisms (Figure 3). Unfortunately, no microbiological cultures of the biopsy were done. The patient then was referred to the department of infectious disease for further evaluation. During the focused anamnesis, the patient reported having recently made several business travels to Africa, Central America, South America and the Caribbean. He had no weight loss, fever, diarrhea, or skin lesions and there were no significant findings on physical examination. A Mantoux test was performed which was negative. The following serological studies were carried out: hepatitis A virus; Epstein-Barr virus; *Trypanosoma cruzi*; cytomegalovirus; Ac HBs; Ag HBs; Ac HBc T; HIV 1 + 2; *Toxoplasma gondii*; *Entamoeba histolytica*; *Echinococcus granulosus*; *Taenia solium* (IgG); Leishmania spp.; strongyloides and schistosoma. All results were negative and do not showed acute infection, except schistosoma with IgM and IgG positive.

Based on the results obtained in the serological tests, a schistosoma infestation (schistosomiasis) was diagnosed and praziquantel was prescribed. During the follow-up the patient referred remission of the abdominal pain and a CT control one year later showed a shrink in size of the paraaortic lesion.

## Discussion

The retroperitoneum is the part of the abdominal cavity that lies between the posterior parietal peritoneum and transversalis fascia. The majority of retroperitoneal masses arise from retroperitoneal organs and are therefore not considered primary retroperitoneal masses [6]. Primary retroperitoneal masses are categorized as solid or cystic (Table 1), depending on their appearance on imaging. Computed tomography (CT) and magnetic resonance imaging (MRI) constitute the mainstay of evaluation (Table 2) [2].

**Table 1:** differential diagnosis of primary retroperitoneal masses

Solid	Neoplastic	Lymphoid tumors
		Lymphoma
		**Sarcomas**
		Liposarcoma
		Malignant fibroushistiocytoma
		Leiomyosarcoma
		**Neurogenictumors**
		Schwannoma
		Paraganglioma
		Ganglioneuroma
		Neurofibroma
		**Immature teratomas**
	**Non-neoplastic**	**Retroperitoneal fibrosis**
		Extramedullary hematopoiesis
		Erdheim-Chester disease
**Cystic**	**Neoplastic**	**Mature teratomas**
		Mucinous cystadenomas
		Cystic mesotheliomas
	**Non-neoplastic**	**Lymphangiomas**
		Müllerian cysts
		Epidermoid cysts
		Pancreatic pseudocysts
		Lymphoceles
		Urinomas
		Hematomas

**Table 2:** differential diagnosis of retroperitoneal masses by the imaging characteristics

Mantle-like primary retroperitoneal masses	Lymphoma
	Metastatic lymphadenopathy Retroperitoneal fibrosis
	Erdheim-Chester disease
**Fat-containing primary retroperitoneal masses**	**Lipoma**
	Well-differentiated liposarcoma dedifferentiated liposarcoma
	Teratoma
**Primary retroperitoneal masses containing myxoid stroma**	**Myxoid liposarcoma**
	Neurofibroma
	Myxoid malignant fibrous histiocytoma
**Primary retroperitoneal masses with calcification**	**Malignant fibrous histiocytoma**
	Teratoma
	Dedifferentiated liposarcoma paraganglioma Ganglioneuroma
**Hypervascular primary retroperitoneal masses**	**Paraganglioma**
	Leiomyosarcoma
	Malignant fibroushistiocytoma
	Other sarcomas

The clinical manifestations of retroperitoneal masses are nonspecific, depending on their location and relation with the adjacent structures. These masses are usually diagnosed as an asymptomatic incidental impalpable tumor [7]. Although the history and physical examination may give a clue for the diagnosis, most retroperitoneal lesions will require further careful investigations, as was in our case where a diagnosis of paraganglioma was first suspected and referred to the endocrinology clinic.

Paraganglioma is a rare NET [8,9]. On cross sectional imaging the appearance of extra adrenal retroperitoneal paraganglioma can overlap those of other tumors of the retroperitoneum and therefore this rare diagnosis must be kept in mind as a differential with retroperitoneal tumors [10]. Although paragangliomas can arise from anywhere along the abdominal paraaortic region, the preponderant site is around the origin of the inferior mesenteric artery, where the collection of paraganglionic tissue is known as the organs of Zuckerkandl [11].

Biochemical testing of urinary and/or plasma fractionated metanephrines and catecholamines is indicated for all paragangliomas, even if clinically non-functional [12]. Radiologic imaging is an important component of assessment being the most commonly used tests: CT, MRI, radioisotope imaging using MIBG, positron emission tomography (PET), octreoscan, and integrated PET/CT [13,14]. In our case, the CT findings of the retroperitoneal lesion (Figure 1A) pointed out a paraganglioma as the first possibility, however, urinary fractionated metanephrines and catecholamines were negative as were also the radioisotope imaging using MIBG and the octreoscan (Figure 2).

Due to the inconclusive results of the workup, the final pathology could only be confirmed with histological examination. There is a concern regarding core biopsies of retroperitoneal masses. A biopsy is contraindicated in a patient suspected of having paraganglioma unless the results of biochemical screening for catecholamine secretion are first negative or the patient is prepared with alpha-adrenergic blockade, because otherwise it can cause severe hypertension from catecholamine crisis. In patients suspecting malignant tumors was though that biopsies of these lesions led to frequent seeding of the tumors, but more recent studies confirm that this risk is extremely low [15,16]. A core biopsy performed via a posterior approach is generally very safe, which was performed to our patient (Figure 1B). After the histology examination and the serological tests, a pseudotumoral form of schistosomiasis was considered as the diagnosis of the retroperitoneal mass.

Schistosomiasis is the second most frequent parasitosis after malaria. The three major species are *Schistosoma mansoni, S. japonicum* and *S. haematobium*. The first two cause intestinal tract disease, while *S. haematobium* causes genitourinary tract disease [4]. Most infected individuals do not develop symptomatic illness. The natural course of the infection depends on the age of primary exposure, the intensity of ongoing exposure, development of immunity against repeat infection and genetic susceptibility [17].

Manifestations of acute infection are generally observed among individuals not living in endemic areas, such as travelers. Manifestations of chronic infection are generally observed among individuals with ongoing exposure in endemic regions. Disease is caused by the host immune response to migrating eggs leading to an eosinophilic granulomatous reaction [18]. Diagnostic evaluation is warranted for patients with clinical manifestations suggestive of schistosomiasis in the setting of appropriate epidemiologic exposure and the approach to diagnosis for returned travelers differs from the approach to diagnosis in endemic settings. Among returned travelers, serology is the most useful test, but it does not reflect definitive evidence of ongoing infection [19]. Among individuals living in endemic areas, the parasite burden should be determined by microscopy for egg detection and antigen detection. Microscopy and polymerase chain reaction assays are used to determine the infecting species. Biopsy is useful as a diagnostic tool in the setting of ectopic disease manifestations and in the absence of demonstrative laboratory diagnostic tools [19].

The pseudotumoral form of *Schistosoma mansoni* is infrequent and can clinically simulates malignant neoplasms. The preferential site is the large intestine, at the level of the sigmoid colon and of the rectum (over 60% of cases) [5]. The most frequent histological picture is characterized by the formation of a large number of granulomas, isolated or confluent, most in the productive phase or in the healing phase by fibrosis, around eggs of *Schistosoma mansoni*. Another type is characterized by a diffuse fibrosis associated or not with the large number of eggs of *Schistosoma mansoni*, usually calcified, with a little tendency to form granulomas [20]. Treatment of schistosomiasis serves three purposes: reversing acute or early chronic disease, preventing complications associated with chronic infection and preventing neuroschistosomiasis. The usual treatment is oxamniquine (hydroxyquinoline derivative) and praziquantel (pyrazinoisoquinoline derivative) [19].

## Conclusion

The finding of a retroperitoneal mass presents many challenges not least of which are the relative rarity and the myriad of diagnostic possibilities. Careful analysis of the history and physical examination may give a clue for the diagnosis. However, most retroperitoneal lesions will not have an obvious diagnosis based on clinical presentation, and will require further careful investigations. Serum catecholamines or tumor markers may point to a specific tumor but good quality imaging either with contrast-enhanced computed tomography scans or magnetic resonance imaging may reveal a characteristic pattern that points to a clear diagnosis. In many cases only the histology examination will provide the final pathology.

## References

[1] Scali EP, Chandler TM, Heffernan EJ, Coyle J, Harris AC, Chang SD (2015). Primary retroperitoneal masses: what is the differential diagnosis. Abdom Imaging.

[2] Mota MMDS, Bezerra ROF, Garcia MRT (2018). Practical approach to primary retroperitoneal masses in adults. Radiol Bras.

[3] Dehal A, Kim S, Ali A, Walbolt T (2015). Primary epithelial neuroendocrine tumors of the retroperitoneum. Perm J.

[4] Nelwan ML (2019). Schistosomiasis: life cycle, diagnosis and control. Curr Ther Res Clin Exp.

[5] Raso P, Hartrung-Toppa N A-MJ (2017). Pseudotumoral intestinal and peritonial form of schistomiasis mansoni in humans: collagen types: retrospective study of 15 cases, literature review. Rev Med Minas Gerais.

[6] Brennan C, Kajal D, Khalili K, Ghai S (2014). Solid malignant retroperitoneal masses-a pictorial review. Insights Imaging.

[7] Elias J, Muglia VF (2019). Magnetic resonance imaging of the perirenal space and retroperitoneum. Magn Reson Imaging Clin N Am.

[8] Neumann HPH, Young WF, Eng C (2019). Pheochromocytoma and paraganglioma. N Engl J Med.

[9] Fishbein L (2016). Pheochromocytoma and paraganglioma: genetics, diagnosis and treatment. Hematol Oncol Clin North Am.

[10] Wijeratne R, Gopal K (2007). Retroperitoneal paraganglioma. Eurorad.

[11] Parmar K, Chandna A, Kumar S (2019). Retroperitoneal paraganglioma: a chameleon masquerading as an adrenal pheochromocytoma. Ann R Coll Surg Engl.

[12] Shen Y, Cheng L, Mariani-Costantini R (2019). Biochemical diagnosis of pheochromocytoma and paraganglioma. Paraganglioma: a multidisciplinary approach.

[13] Mercado-Asis LB, Wolf KI, Jochmanova I, Taïeb D (2018). Pheochromocytoma: a genetic and diagnostic update. Endocr Pract.

[14] Kroiss AS (2019). Current status of functional imaging in neuroblastoma, pheochromocytoma and paraganglioma disease. Wien Med Wochenschr.

[15] Hwang SY, Warrier S, Thompson S, Davidson T, Yang JL, Crowe P (2016). Safety and accuracy of core biopsy in retroperitoneal sarcomas. Asia Pac J Clin Oncol.

[16] Crowe P (2018). A retroperitoneal mass needs respect. ANZ J Surg.

[17] Costain AH, MacDonald AS, Smits HH (2018). Schistosome egg migration: mechanisms, pathogenesis and host immune responses. Front Immunol.

[18] McManus DP, Dunne DW, Sacko M, Utzinger J, Vennervald BJ, Zhou XN (2018). Schistosomiasis. Nat Rev Dis Prim.

[19] Soentjens P, Clerinx J (2019). Schistosomiasis: diagnosis. UpToDate.

[20] Schwartz C, Fallon PG (2018). Schistosoma “eggs-iting” the host: granuloma formation and egg excretion. Front Immunol.

